# Abnormal Motor Activity and Thermoregulation in a Schizophrenia Rat Model for Translational Science

**DOI:** 10.1371/journal.pone.0143751

**Published:** 2015-12-02

**Authors:** Gyongyi Horvath, Gabriella Kekesi, Zita Petrovszki, Gyorgy Benedek

**Affiliations:** 1 Department of Physiology, Faculty of Medicine, University of Szeged, Szeged, Hungary; 2 Institute of Physical Education and Sport Medicine, Juhász Gyula Faculty of Education, University of Szeged, Szeged, Hungary; University of Cincinnati, UNITED STATES

## Abstract

**Background:**

Schizophrenia is accompanied by altered motor activity and abnormal thermoregulation; therefore, the presence of these symptoms can enhance the face validity of a schizophrenia animal model. The goal was to characterize these parameters in freely moving condition of a new substrain of rats showing several schizophrenia-related alterations.

**Methods:**

Male Wistar rats were used: the new substrain housed individually (for four weeks) and treated subchronically with ketamine, and naive animals without any manipulations. Adult animals were implanted with E-Mitter transponders intraabdominally to record body temperature and locomotor activity continuously. The circadian rhythm of these parameters and the acute effects of changes in light conditions were analyzed under undisturbed circumstances, and the effects of different interventions (handling, bed changing or intraperitoneal vehicle injection) were also determined.

**Results:**

Decreased motor activity with fragmented pattern was observed in the new substrain. However, these animals had higher body temperature during the active phase, and they showed wider range of its alterations, too. The changes in light conditions and different interventions produced blunted hyperactivity and altered body temperature responses in the new substrain. Poincaré plot analysis of body temperature revealed enhanced short- and long-term variabilities during the active phase compared to the inactive phase in both groups. Furthermore, the new substrain showed increased short- and long-term variabilities with lower degree of asymmetry suggesting autonomic dysregulation.

**Conclusions:**

In summary, the new substrain with schizophrenia-related phenomena showed disturbed motor activity and thermoregulation suggesting that these objectively determined parameters can be biomarkers in translational research.

## Introduction

Animal models are not only important tools for understanding pathological mechanisms, but they are also necessary for testing hypotheses that cannot be addressed in human studies and for developing and preclinical testing of new treatments [1]. However, investigating psychiatric illnesses in animals is quite difficult, as these illnesses are characterized by disturbances in functions assigned to humans only (e.g., hallucinations). Nevertheless, certain aspects of schizophrenia can indeed be modeled in animals. It is well-known that both genetic predisposition and environmental factors contribute to the pathomechanism of this neuropsychiatric disorder; therefore, the combination of these interventions may lead to a reliable chronic schizophrenia model with relatively high constructive validity. Recently a complex animal model has been developed by selective breeding based on behavioral alterations after combined subchronic ketamine treatment (NMDA-receptor antagonist) and postweaning social isolation [2,3]. These animals showed several signs of schizophrenia, i.e., disturbed pain sensitivity, sensory gating, stereotypic behaviors and cognitive functions suggesting that they might be a more reliable model of schizophrenia than naive animals with isolation and ketamine treatment or the new substrain without any treatment.

Impairment in motor behaviors is an important sign of schizophrenia, as it is associated with its subtype, psychopathology and medication; thus, excessive motor agitation or reduced motor activity, even akinetic episodes, could be observed [4,5]. Motor activity changes in different schizophrenia animal models have been observed mainly in open field for a short period [6], and few animal data are available about the motor activity changes in schizophrenia rodent models in freely moving condition for a long period [7,8].

A body of evidence has demonstrated disturbed thermoregulation in schizophrenic patients with controversial results, but its mechanism has not been revealed [9–11]. Only few studies have investigated the body temperature (BT) changes under restrained circumstances in schizophrenic animal models, but no data are available about the thermoregulation during freely moving, unstressed conditions [12,13]. Therefore, our objective was to reveal 1) the circadian rhythm of thermoregulation and motor activity in the 18^th^ generation of the new substrain after social isolation and ketamine treatment in freely moving conditions, and 2) the effects of changes in light condition and different interventions (change of bedding, handling or intraperitoneal (IP) vehicle injection) on these parameters as well. One valuable tool to determine the variability of some physiological data is the Poincaré plot analysis (PPA) which has been applied for heart rate, blood pressure and electroencephalogram data [14–17]. This time-domain analysis can determine short- and long-term variabilities and asymmetry in the variabilities. While the variabilities in BT have also been investigated in a few studies, no PPA has been performed yet for this parameter [18]. The further goal was to reveal the circadian rhythm of these Poincaré parameters in control animals and to disclose the potential disturbances in these factors of the new substrain.

## Materials and Methods

All experiments were carried out with the approval of the Hungarian Ethical Committee for Animal Research (Reference number: XIV/03285/2011).

### 2.1 Selective breeding process

The paradigm for selective breeding has previously been described [2]. Briefly: Wistar rats, after weaning at 3 weeks of age, were tested with the tail-flick (TF) test (48°C hot water) to assess pain sensitivity and then housed individually for 28 days. The animals were treated with ketamine (Calypsol, Gedeon Richter Plc., Budapest, Hungary; 30mg/kg IP, 4mL/kg, daily, 5 times/week, 15 injections in total) from 5 to 7 weeks of age. Then the animals were re-housed (4–5/cage), and 1 week of recovery followed with no treatment. Starting at the age of 9 weeks, the pain sensitivity with TF test, the sensory gating with prepulse inhibition (PPI), and the cognitive functions and stereotypic behavior on hole-board (HB) test were assessed (Table 1). Animals with the highest level of disturbances in these parameters were used for selective breeding throughout 18 generations [2,3].

**Table 1:** Schedule of the experimental protocol for selective breeding and behavioral testing.

	Weaning	Procedures for selective breeding			Behavioral testing		
Age (weeks)	3	4	5–7	8	9	10	16–24
**Naive rats**	TF, PPI		group housing (3–4 rats/cage)		TF, PPI	HB	Telemetry recordings
**New substrain**		social isolation	social isolation + ketamine treatment (30 mg/kg/daily)	group housing			

Abbreviations: TF: tail-flick test, PPI: prepulse inhibition test, HB: hole-board test.

### 2.2 Telemetric experiments

Two experimental groups of 6-6 rats were compared: naive socialized male rats without ketamine treatment; and the 18^th^ generation of selectively bred male rats with social isolation and ketamine treatment. After the abovementioned behavioral tests (Table 1), the animals were involved in the telemetric experiments between 4 and 6 months of age. E-Mitter system with battery-free and implantable transponders attached to PC-based data acquisition software is appropriate to monitor abdominal temperature (°C) and gross locomotor activity (in arbitrary unit) in freely moving animals in their home cage with 1 min of sampling frequency (Starr Life Sciences, E-Mitter, Vitalview, Oakmont, PA, USA). Animals were intraabdominally implanted with E-Mitter transponders under ketamine-xylazine (72 and 8mg/kg IP, respectively) anesthesia to minimize the suffering of animals during the surgery and post-surgery recovery. Following the surgical procedure the animals were injected with gentamycin (10 mg/kg, subcutaneously) to prevent infection and were housed individually in cages (42x30x19cm) with *ad libitum* access of chow and tap water. The cages were placed on receiver platforms in an isolated room (23°C and 66% humidity) with a 6:00 a.m.–6:00 p.m. light cycle. After 5 days of recovery period, BT and motor activity were monitored continuously for 13 days. The animals were not disturbed for 8 days in total. Each of them was handled (for about 1 min) and bed-changed (which means handling together with new environment) on 2–2 different days, and IP vehicle injections were applied at once between 8:30 and 9:00 a.m. Since the intraindividual variabilities of both motor activity and BT were high, more data sampling was required. To do this, the data of different days with the same conditions were pooled as applied in an earlier study [19]; thus, 48 recordings (6 animals for 8 days) without any disturbances and 30 recordings (6 animals for 5 days) with disturbances (handling, bed-change and/or vehicle injection) were pooled, but they were separately analyzed for dark and light phases. Regarding the analysis of motor activity and BT, several parameters were calculated for the active (dark) and passive (light) phases without any intervention, as indicated in Table 2 [20]. To observe the acute effects of changes in light conditions or different interventions (handling, bed changing or IP vehicle injection), the mean of activity level (AL) and BT per 10 min epoch for 1 or 2 hours after the actions were analyzed (based on a preliminary study), and the baseline value was calculated as the mean of values for an hour before the actions (Table 2).

**Table 2:** Parameters used to describe motor activity (a) and body temperature (b).

***a*. *Motor activity***		
Activity level (AL)		the mean number of activity counts per 1 hour epoch
Movement index (MI)		the percentage of 1 min epochs with an activity count >0
Relative movement index (RMI)		the mean number of activity count during 1 min epochs with an activity count >0, reflecting the degree of activity during the active epochs
Phase number (PN)		the total number of inactive and active periods, reflecting the rhythmicity of the motor behavior
Phase length (PL)	DI	the mean duration of uninterrupted immobility periods provides measures of the length of immobility epochs
	DA	the mean duration of uninterrupted active periods provides measures of length of active epochs
**b. Body temperature (BT)**		
mean BT		the mean BT per 1 hour epoch
BTmin and BTmax		minimum and maximum values of BT
BT amplitude		difference of maximum and minimum BT
BT variability	SD1	the short-term variability measures the dispersion of points perpendicular to the line of identity on Poincaré plot
	SD2	the long-term variability measures the dispersion of points along the line of identity on Poincaré plot
BT asymmetry (BTA)	C1d	relative contribution of decreases to short-term variance
	C1i	relative contribution of increases to short-term variance
	C2d	relative contribution of decreases to long-term variance
	C2i	relative contribution of increases to long-term variance

### 2.3 Statistical analysis

#### Poincaré plot analysis

**Fig 1:**
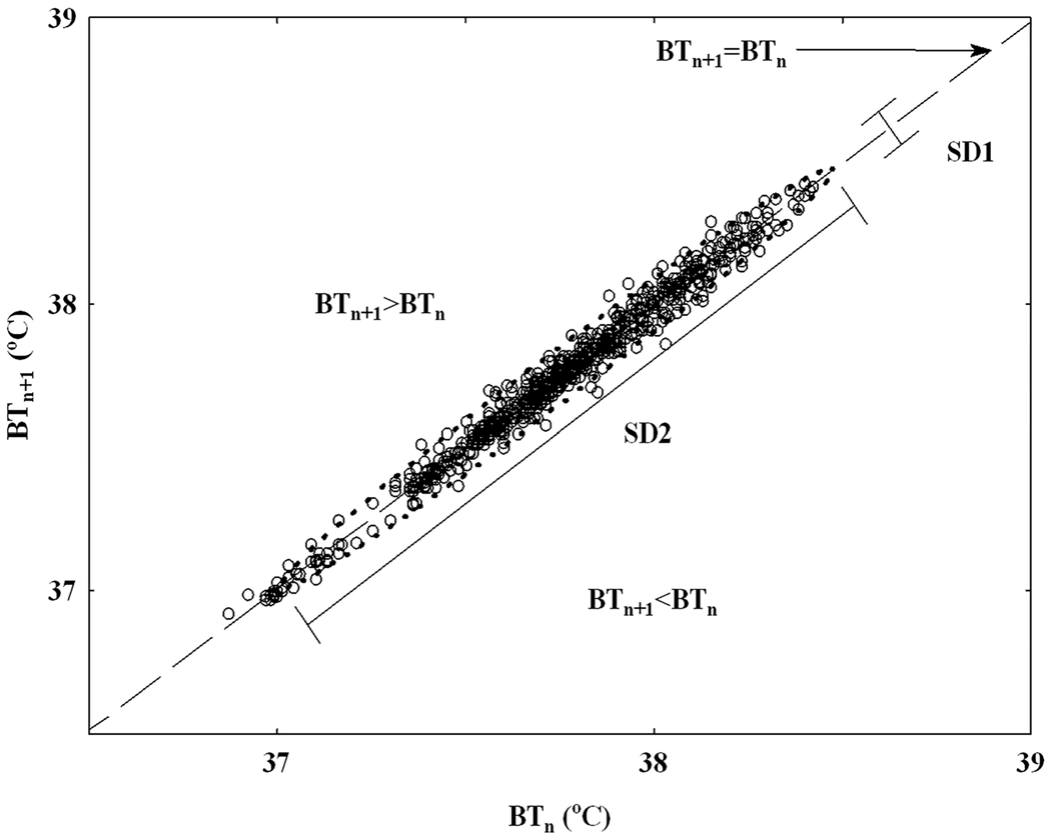
A sample Poincaré plot analysis of temperature time series derived from a 12-h recording during dark phase from a control rat. The dashed line is the identity line; all points on this line correspond to two neighboring temperatures with equal values; all points above this line correspond to BT increases (BT_n+1_>BT_n_); all points below this line correspond to BT reductions (BT_n+1_<BT_n_). BT_n_: BT of the current value, BT_n+1_: BT of the next value. SD1: the standard deviation quantifying the dispersion of points in the Poincaré plot across the identity line. SD2 represents the dispersion along the identity line.

The time domain analysis of temperature variability was performed separately for light and dark periods (containing 720–720 data points during 12–12 hours) to characterize the scatter plot of BT using the Poincaré descriptors. In the PPA, each point has coordinates (BT_n_, BT_n+1_), so that it binds a temperature with the next one to display nonlinear aspects of the data sequence [16,17]. The line of identity is the 45° imaginary diagonal line on the Poincaré plot, and the points falling on this line has the property of BT_n_ = BT_n+1_ (Fig 1). The basic descriptors of the PPA (Table 2b) are the SDNN, which corresponds to the total variability of the temperature time series. SD1 (short-term variability) measures the dispersion of points perpendicular to the line of identity, whereas SD2 (long-term variability) describes the dispersion along the line of identity [16,17]. There is the following relation between these descriptors:

$$SD1^{2}=Var\left(\frac{BT_{n+1}-BT_{n}}{\sqrt{2}}\right)$$ (1a)

$$SD2^{2}=Var\left(\frac{BT_{n+1}-BT_{n}}{\sqrt{2}}\right)$$ (1b)

$$SDNN^{2}=\frac{SD1^{2}+SD2^{2}}{2}$$ (2)

The body temperature asymmetry (BTA) phenomenon reflects the consistently different contribution of temperature decreases and increases to the variance-based descriptors of the variability of BT time series [16,17]. The points located on the identity line have the following property: BT_n_ = BT_n+1_, above it, there are increases (BT_n+1_ >BT_n_), and below this line, there are decreases (BT_n+1_<BT_n_) (Fig 1). To calculate BTA, the short and long term variances were partitioned accordingly:

$$SD1^{2}=SD1_{i}^{2}+SD1_{d}^{2}$$ (3a)

$$SD2^{2}=SD1_{i}^{2}+SD1_{d}^{2}$$ (3b)

After calculation of these parameters, normalized contributions of increases (C1_i_, C2_i_) and decreases (C1_d_, C2_d_) to SD1^2^ or SD2^2^ were partitioned (Table 2b):

$$C1_{i}=\frac{SD1_{i}^{2}}{SD1^{2}}$$ (4a)

$$C1_{d}=\frac{SD1_{d}^{2}}{SD1^{2}}$$ (4b)

$$C2_{i}=\frac{SD2_{i}^{2}}{SD2^{2}}$$ (4c)

$$C2_{d}=\frac{SD2_{d}^{2}}{SD2^{2}}$$ (4d)

Data are expressed as means±S.E.M. Different parameters were compared with one-way or repeated-measures two-way ANOVA; the Newman-Keuls *post hoc* test was also applied. For the asymmetry analysis, the binomial test for comparing the numbers of asymmetric cases and the Wilcoxon test for comparing the relative contributions were used [16]. Statistical analysis was performed with Statistica 12.0 software (Statsoft, Tulsa, Oklahoma, USA). Differences were considered significant for p<0.05.

## Results

In agreement with our recent studies [2,3], the naive and selected rats involved in the telemetric experiments showed significant differences in the behavioral tests. The new substrain showed decreased acute heat pain sensitivity indicated by longer tail-flick latency (13.8±0.92 s) compared to the naive rats (10.7±1.13 s; p<0.05). Regarding the sensory gating of acoustic startle reflex, it decreased close to significantly (p = 0.07) in the new substrain (33.9±7.62%) compared to the naive group (55.7±7.58%). Comparing their cognitive performance in the modified HB test, the selected rats could collect significantly fewer food rewards (1.5±1.19 over 16) compared to their naive counterparts (13.2±1.19 over 16; p<0.01), and it was accompanied by impaired stereotypic behavior (decreased rearing, locomotor and exploratory, but increased self-grooming activities).

### 3.1 Motor activity and body temperature alterations during undisturbed circumstances

**Fig 2:**
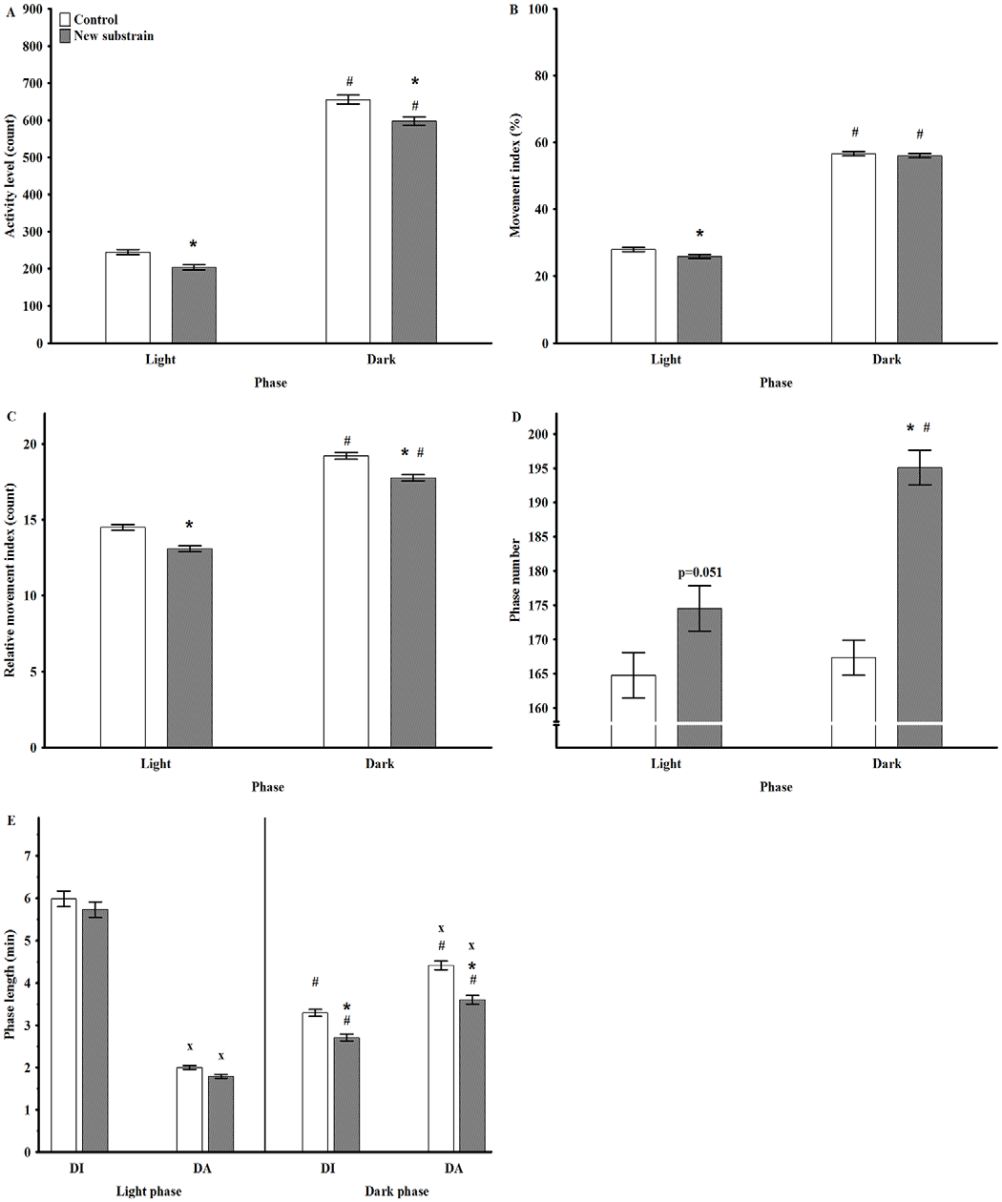
Motor activity parameters in undisturbed circumstances during light and dark phases. Mean AL (activity level; A), MI (movement index; B), RMI (relative movement index; C), PN (phase number; D), PL (phase length; E) with duration of uninterrupted immobility (DI) or active (DA) period. * p<0.05 between groups; # <0.05 between the day cycles; x p <0.05 between the DI and DA. Data are presented as means ± SEM.

**Fig 3:**
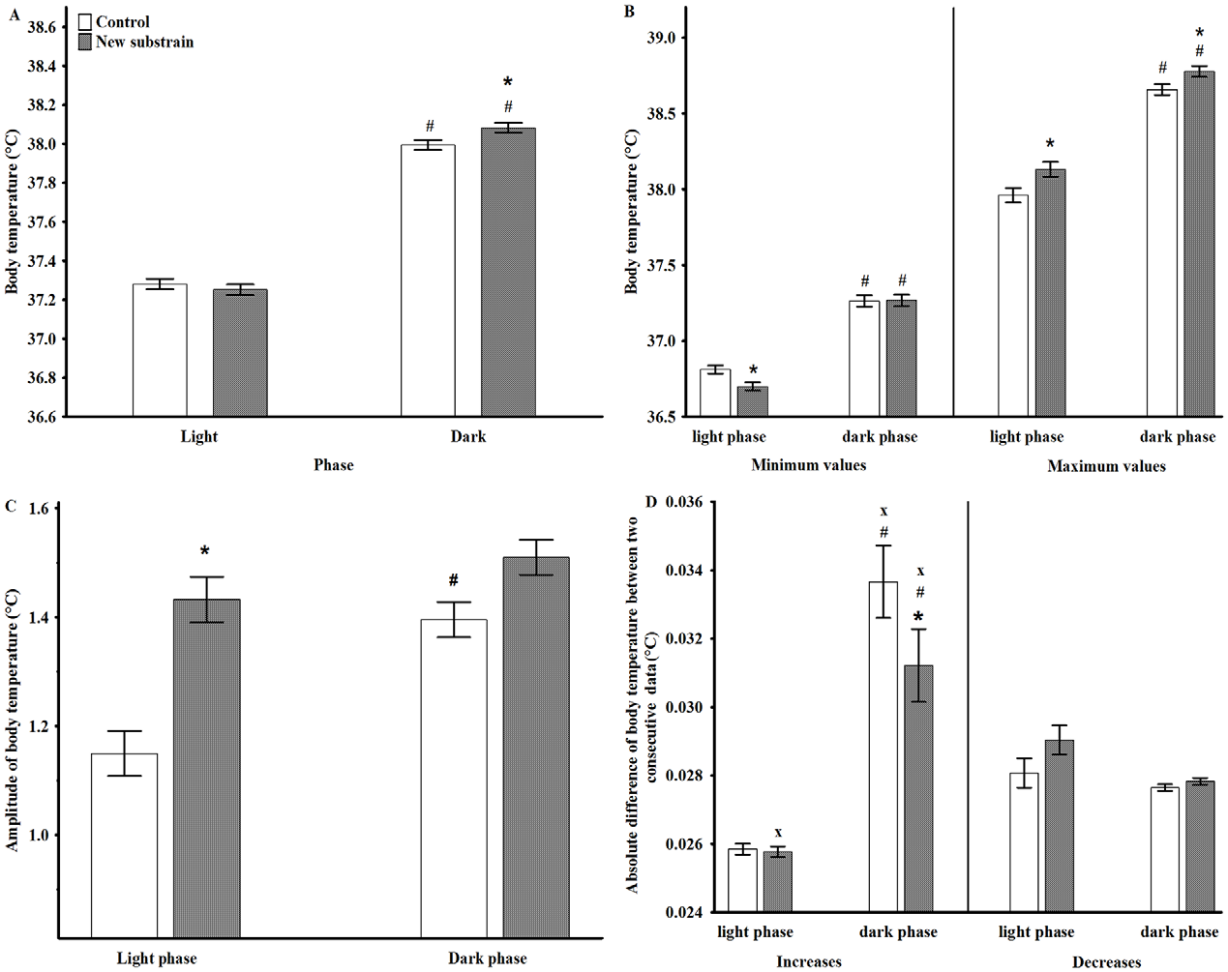
Body temperature parameters in undisturbed circumstances during light and dark phases. The mean values of BT minimum (A), BT maximum (B), BT amplitudes (C), and the mean of changes in BT between two consecutive data points (D). * p <0.05 between groups; # p <0.05 between the day cycles; x p <0.05 between the BT decreases and increases. Data are presented as means ± SEM.

Motor activity and BT did not change significantly during the 8 days, which suggest that these parameters were stable. Motor activity and BT of rats belonging to either of the two groups showed daily rhythm with dark maxima and day minima characteristic of most rodent species (Figs 2A and 3A).

The analysis of AL at dark and light phases showed significant effects of group (F_1,110_ = 32.73, p<0.001), phase (F_1,110_ = 2514, p<0.001) and a close to significant effect of interaction (F_1,110_ = 3.33, p = 0.07; Fig 2A); thus, the animals of the new substrain exhibited decreased AL during both phases compared to control rats. The analysis of mean values of BT showed a significant effect of phase (F_1,110_ = 3929, p<0.001), and phase and group interaction (F_1,110_ = 21.00, p<0.001); thus, the new substrain had significantly higher temperature during the dark period (Fig 3A). Therefore, lower activity was accompanied by higher BT in the new substrain.

The analysis of the movement index (MI) and relative movement index (RMI) (Table 2b) showed that these values were higher during the active phase compared to the inactive one in both groups (Fig 2B and 2C). The new substrain had significantly lower MI during the inactive phase, while RMI decreased in both phases compared to the control group. Thus, these animals had reduced number of active epochs with decreased motor activity during these epochs.

The analysis of phase number (PN) revealed that the animals of the new substrain exhibited significantly higher PN during the active phase compared to the control group and to the passive period (Fig 2D). Regarding the phase differences in phase length (PL), DI (length of immobility epochs) was significantly longer during the passive phase compared to the active one, while DA (length of active epochs) showed the opposite pattern in both groups (Fig 2E); thus, DI was significantly longer than DA during light phase, while the opposite was observed in the dark period. Furthermore, the new substrain exhibited significantly shortened DI and DA during their active period.

Regarding the detailed analysis of ranges of BT (Table 2a), animals in both groups had significantly higher minimum and maximum values during the active phase than in the passive one (Fig 3B). Moreover, the new substrain had significantly lower minimum value during the inactive phase and higher maximum values in both phases compared to control animals. Therefore, the amplitude of BT increased during the dark period compared to the light one in the control group, but the new substrain did not show this pattern since they had significantly higher BT amplitude during the light phase than the control group (Fig 3C).

Regarding the analysis of mean changes in BT between two consecutive data points, the degree of BT increases during the dark phase were significantly larger compared to the light phase or to the BT decreases in both groups (Fig 3D). The comparison of the two groups revealed significantly smaller degree of increases in the new substrain compared to the control animals during the active phase.

### 3.2 The acute effects of changes in light conditions and different interventions

**Fig 4:**
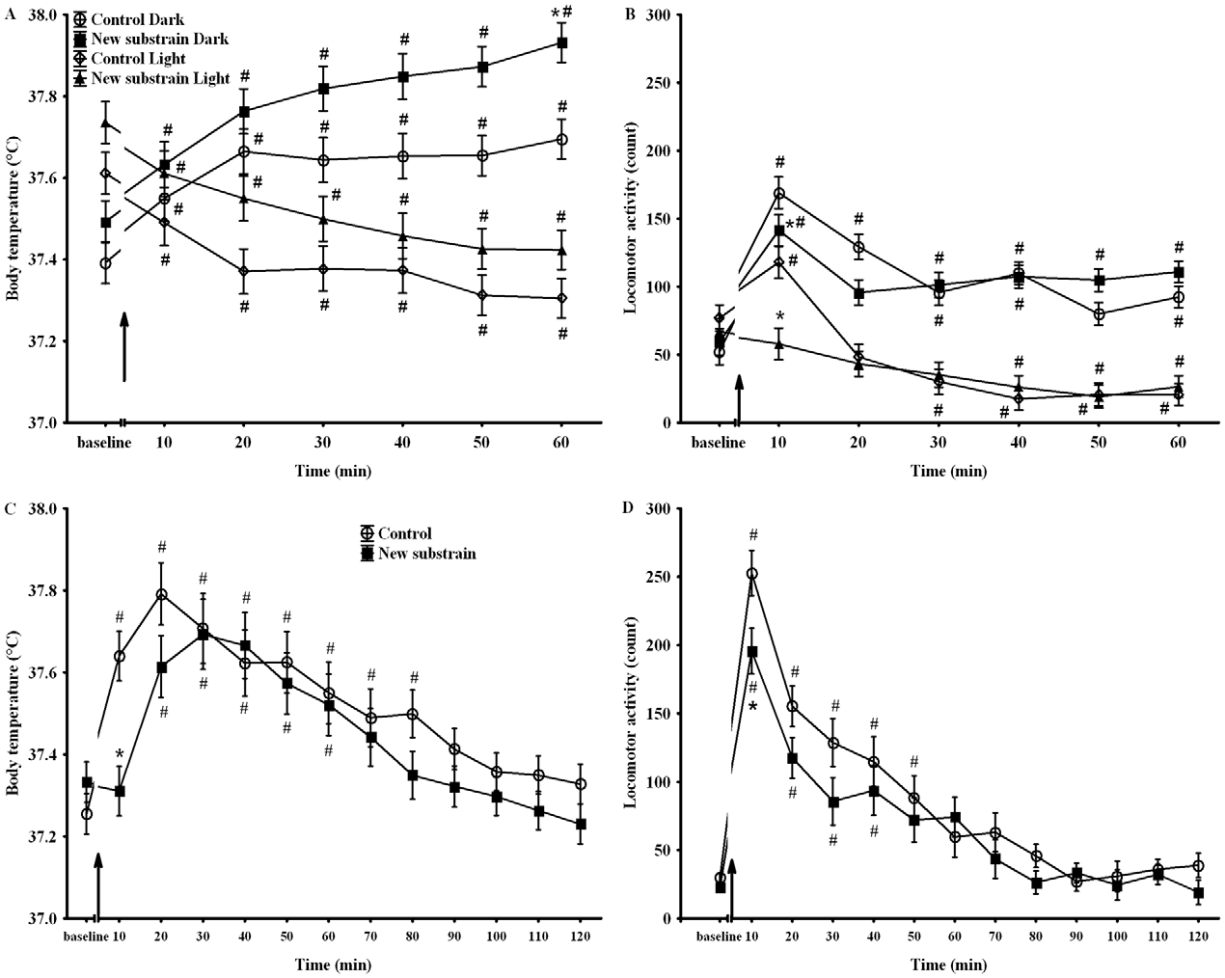
**BT (A) and motor activity (B) alterations during the light-phase changes for an hour.** BT (C) and motor activity (D) changes after interventions for two hours. ↑ signs the beginning of changes in light condition or the interventions. * p <0.05 between groups; # p <0.05 compared to baseline. Data are presented as means ± SEM.

The analysis of BT and motor activity values at the beginning of changes in light conditions revealed that BT of the new substrain was higher in both light on or off conditions, in spite of the fact that the motor activity changes were blunted in this group (Fig 4A and 4B). The different interventions (handling, cleaning or vehicle injection) produced significant, temporary and similar effects. Thus, all the three interventions caused temporary hyperlocomotion accompanied by increased BT in both groups; therefore, these data were pooled for further analysis. The analysis of these data showed that the animals of the new substrain exhibited delayed hyperthermia with decreased hyperactivity (Fig 4C and 4D).

#### 3.2.1 Poincaré plot analysis of BT

The Poincaré descriptors (Table 2b) were analyzed separately for the dark and light periods without disturbances. As Fig 1 shows, the shape of the scatter plot was similar to a slim cigar (SD1 is about 10 times as low as SD2); thus, SD2 had high contribution to SDNN; therefore, it had almost the same value as SD2. For that reason, the results of SDNN were not presented. The very slim shape of the plot indicated strict linearity of the data, which suggests that 1 min was an appropriately short time-lag for BT sampling.

**Fig 5:**
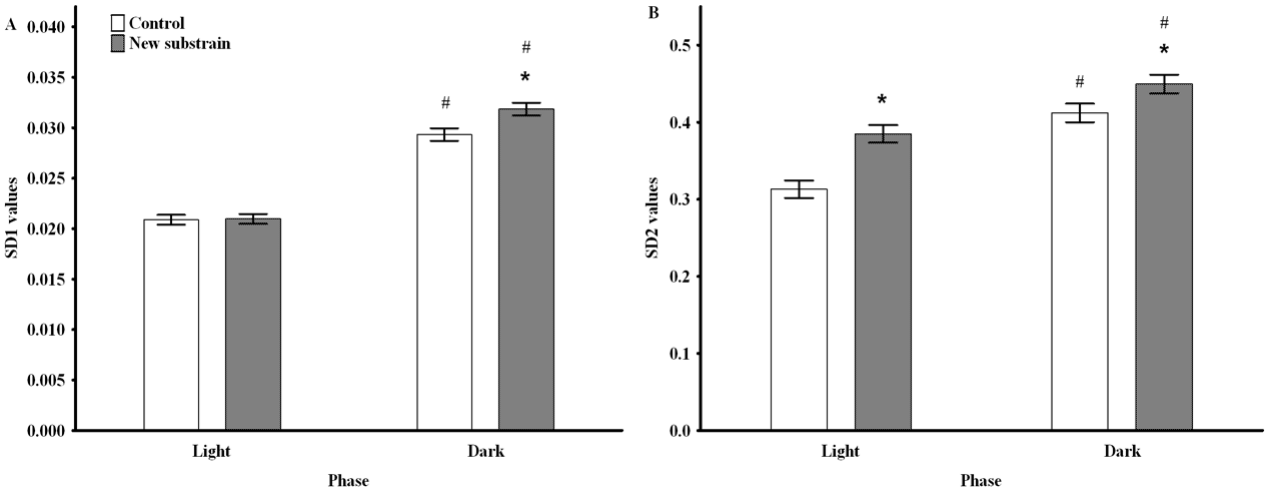
Poincaré plot analysis of body temperature in undisturbed circumstances during light and dark phases. Changes in SD1 (A) and SD2 (B). * p <0.05 between groups; # p <0.05 between the day cycles. Data are presented as means ± SEM.

Both SD1 and SD2 were significantly larger in both groups during the active phase compared to the passive period (Fig 5B and 5C). Furthermore, the new substrain had increased short-lasting variability during the active phase, but higher long-lasting variability during both phases than the control rats. Regarding the short-term asymmetry of temperature, the number of SD1d was dominant, and the mean CD1d was significantly larger than SD1i in both groups independently of the day phase (Table 3). Thus, the short-term asymmetry was present in the two groups during both day phases with a significantly lower level in the new substrain (Table 3). The comparison of the short-term asymmetry of dark vs. light periods revealed that the active phase was accompanied by decreased asymmetry in the control group but not in the new substrain.

**Table 3:** The short- and long-term asymmetries of body temperature.

	Short-term asymmetry		Long-term asymmetry	
	N (%) of SD1d>SD1i	C1d	N (%) of SD2d<SD2i	C2i
**Light phase**				
**control**	44 (91.7%)*	0.577*	43 (89.6%)*	0.537*
**new substrain**	37 (77.1%)*	0.525* #	31 (64.4%)	0.510#
**Dark phase**				
**control**	37 (77.1%)*	0.525* +	34 (70.8%)*	0.525*
**new substrain**	29 (60.4%)	0.512* #	40 (83.3%)*	0.525*

The number of cases (N) have C1d > C1i, or C2d < C2i, and the degree of the relative contribution of decreases (C1d) or increases (C2i) to short- and long-term asymmetry, respectively.

* Significant degree of asymmetry;

^#^ p<0.05 compared to control group;

^+^ p<0.05 compared to light phase.

Regarding the long-term asymmetry of temperature, the number of SD2i was dominant, and the mean SD2i was significantly larger than SD2d in the control group in both day phases, which reflects significant asymmetry. In contrast, the new substrain had significant long-term asymmetry only during the light phase (Table 3). Thus, the degree of the long-term asymmetry was clearly decreased in the new substrain compared to the control group during the light, but not the dark phase. The comparison of the long-term asymmetry of dark vs. light periods did not show significant differences in either group.

## Discussion

Telemetric investigation revealed that the new substrain had significant alterations in thermoregulation and locomotor activity registered for a long period under unrestrained conditions. Thus, they had lower activity and disturbed rhythmicity (enhanced PN with decreased PL) accompanied by higher value and enhanced amplitude of BT. PPA demonstrated significant differences in the BT variabilities and BTA between the light and dark phases in both animal groups. The new substrain had higher degree of short- and long-term variabilities but lower level of asymmetry compared to the control group.

Altered motor activity in schizophrenia constitutes an integral part of its psychopathology; therefore, motor symptoms are used as a diagnostic criterion [4,5,21]. Disturbances in motor activity and its circadian rhythm have been studied in schizophrenic patients with actigraphy showing that the total motor activity was lower in patients compared to control subjects [5,21,22]. Several chronic schizophrenia animal models show changes in motor activities; however, most of the studies used short observation periods in open field [6]. Slight changes in the activity were found on hole-board test in our new substrain suggesting that the brief investigation period could not reveal the fine disturbances in motor activity [3]. The telemetry method allows frequent measurements of different physiological parameters in rodents without inducing stress-related changes [23,24]. Few studies have investigated the locomotor activity in unstressed conditions for long-term period in schizophrenia animal models with controversial results [7,8,25]. In agreement with mutant mouse models [8,26], this new substrain of rats had decreased motor activity with abnormal (saccadic) rhythmicity revealing disrupted architecture of motor behavior. It is well-known that schizophrenic patients show disrupted circadian activity rhythms and sleep-wake patterns including higher nocturnal movement and a less clear definition of activity onset in the morning and offset in the evening [21,22,26]. In agreement with these human studies, changes in light condition or different interventions caused blunted motor responses in the new substrain, too.

The maintenance of BT within narrow limits is a homeostatic control that is critical for survival; therefore, BT is one of the most commonly studied variables as a powerful indicator of circadian synchrony [27]. Abnormal thermoregulation has been reported in schizophrenic patients; thus, drug-free schizophrenic patients exhibit elevated BT both in resting state and during exercise, and it has correlated with their psychiatric rating scale score [10,28]. No data are available about the BT changes in schizophrenia animal models measured in unrestrained condition. We observed that in spite of the significantly lower activity during both phases in the new substrain; these animals had higher BT with higher maximum values. One possible explanation for this phenomenon might be that the saccadic rhythmicity of the motor activity masked the effect of hypoactivity on thermoregulation, even leading to hyperthermia during the active phase. However, the minimum temperature was lower in the passive period in the new substrain compared to the control group; thus, the prolonged inactivity led to enhanced hypothermia in this group. All of these alterations support a large scale of disturbances in the thermoregulation of the new substrain.

PPA is a classical nonlinear analytical method that can analyze the variability of time series signals. The few studies that have applied fast Fourier or cosinor analysis suggest that variability in BT can be implicated for the diagnosis of cardiovascular disorders [18]. Poincaré plot indices have advantages because the data have no requirements for normal distribution or special processing that spectral analysis requires, but it provides a lot of information, especially if the position of the points relative to the line of identity is taken into consideration [14,16]. Our data first showed that circadian differences in the SD1 and SD2 of BT were consistent findings in control rats, i.e., significantly higher degree of short- and long-term variabilities were observed during the active phase compared to the passive one, which was in agreement with the trend in the heart rate variability in healthy and schizophrenic subjects [29]. Asymmetry in BT was also observed, i.e., lower contribution of BT increase to short-term variability, and higher contribution of BT increase to long-term variability were detected in agreement with the pattern of the asymmetry of heart rate [16,19]. The degree of these asymmetries depended on day phases, and the new substrain had higher level of variabilities with blunted asymmetry. Asymmetry is present in physiological systems as it is a fundamental property of the non-equilibrium system, and it is related with nonlinear dynamics and time irreversibility exhibiting complex inter-relationships [30,31]. It has been shown that heart rate asymmetry is decreased with pathology, thus providing a marker for any loss of normal functionality [17,30]. The blunted asymmetry of BT may indicate pathological abnormalities in the thermoregulation of the new substrain, which suggests that it has a more chaotic pattern. A number of mechanisms might be behind these alterations including somatic, autonomic and/or hormonal changes. It can be proposed that besides the locomotor alterations, autonomic dysregulation (e.g., increased sympathetic tone) might also have taken part in the development of hyperthermia, and its enhanced variability and decreased asymmetry observed in the new substrain [32]; however, further experiments are required to reveal the possible changes in the activity of the autonomic nervous system in the new substrain. Several data have shown decreased heart rate variability in schizophrenic patients, which might be due to the enhanced sympathetic and decreased parasympathetic tones [29,33–35], but further human studies would be required to reveal whether there are similar changes in the BT parameters, too. Schizophrenia is a disease characterized by complex disturbances in multiple neurotransmitter systems including the abnormalities in both the dopaminergic and the glutamatergic systems [36,37]. These transmitters are also involved in the thermoregulation, motor activity and their circadian rhythm; therefore, the abnormalities in these parameters are not unexpected sings of schizophrenia [28,38–41]. Further studies are necessary to clarify the possible molecular biological and/or genetic backgrounds of these alterations to enhance predictive validity of this model.

### Limitations

It is important to note that this telemetry device required an unphysiological environment for rats, which means that the animals were kept alone in their cages. Sleep–wake cycles in individuals with schizophrenia ranged from well entrained to highly disturbed rhythms with fragmented sleep epochs, together with delayed melatonin onsets and higher levels of daytime sleepiness [42]. Further electroencephalography experiments are required to reveal the possible abnormalities in the sleep–wake cycles of these animals.

Regarding the construct validity of our model, it is important to mention that naive animals after social isolation and ketamine treatment showed some alterations related to schizophrenia [43,44]; furthermore, the combination of these environmental factors with genetic susceptibility through several generations led to higher reliability of the investigated signs in later generations [2,3]. All the behavioral traits seemed to be inheritable, which suggests that this might be a reliable and practical animal model for schizophrenia. However, based on these results, it cannot be decided whether these deficits reflect primary disease-related deficits in discrete brain regions or simply secondary symptoms or just generalized functional impairments. Therefore, further molecular–biological studies are required to determine the changes in the receptor binding activity of different neurotransmitters (e.g., dopamine, glutamate). Additionally, characterization of responses of the observed behavioral deficits to antipsychotic treatment may provide further evidence for the predictive validity of our model.

### Conclusions

In conclusion, the data give strong argument to support the use of telemetry as a complementary tool to evaluate BT and motor activity changes of rats in freely moving condition. These data show that the new substrain has shown prolonged alterations in motor behavior, thermoregulation, and circadian rhythm after juvenile social isolation and ketamine treatment, in accordance, at least partially, with the clinical picture of schizophrenic patients. We suppose that the presence of these symptoms besides other behavioral abnormalities enhanced the face validity of this schizophrenia animal model. These results first characterized the pattern of temperature variability and asymmetry with PPA in control animals, and it revealed autonomic dysregulation in a schizophrenia animal model. The precise practical use of these results has yet to be found, but it seems that the analysis of variabilities and asymmetric patterns may contribute to our knowledge and a better understanding of BT physiology.
